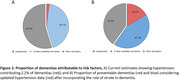# Hypertension's underestimated role in dementia

**DOI:** 10.1002/alz70860_105645

**Published:** 2025-12-23

**Authors:** Abolfazl Avan, Vladimir Hachinski

**Affiliations:** ^1^ University of Western Ontario, London, ON, Canada

## Abstract

**Background:**

Hypertension's role in dementia is regarded as modest, because it often overlooks stroke's role. We assessed the dementia risk attributed to hypertension, considering stroke as an intermediary factor, given that stroke is associated with an increased dementia risk and that over half of strokes are linked to hypertension.

**Method:**

We recalculated hazard ratios and the dementia risk proportion attributable to hypertension using weighted population attributable fractions (accounting for overlapping risk factors), incorporating the dementia risk associated with stroke, the stroke risk linked to hypertension, and the prevalence of hypertension.

**Result:**

Our analysis reveals that hypertension increases the risk of stroke fivefold and the risk of dementia eightfold. This corresponds to approximately 16.6% of dementia cases being preventable through hypertension control only (Figure 1). By addressing hypertension effectively, the overall potential for dementia prevention could exceed 59.6%.

**Conclusion:**

Our recalculations of current evidence suggest that hypertension's role in dementia could be eight times higher than previous estimates when accounting for the role of stroke, underscoring the urgent need for enhanced global prevention strategies. Hypertension's high lifetime risk, widespread prevalence, frequent underdiagnosis, and inadequate management offer the single greatest opportunity for delaying, allaying, or preventing stroke, heart disease, and dementia.